# An imbalance in autophagy contributes to retinal damage in a rat model of oxygen‐induced retinopathy

**DOI:** 10.1111/jcmm.16977

**Published:** 2021-10-08

**Authors:** Noemi Anna Pesce, Alessio Canovai, Flavia Plastino, Emma Lardner, Anders Kvanta, Maurizio Cammalleri, Helder André, Massimo Dal Monte

**Affiliations:** ^1^ Department of Biology University of Pisa Pisa Italy; ^2^ Department of Clinical Neuroscience Division of Eye and Vision St Erik Eye Hospital Karolinska Institutet Solna Sweden

**Keywords:** autophagy, cell death, necroptosis, oxygen‐induced retinopathy, rat model, retinopathy of prematurity

## Abstract

In retinopathy of prematurity (ROP), the abnormal retinal neovascularization is often accompanied by retinal neuronal dysfunction. Here, a rat model of oxygen‐induced retinopathy (OIR), which mimics the ROP disease, was used to investigate changes in the expression of key mediators of autophagy and markers of cell death in the rat retina. In addition, rats were treated from birth to postnatal day 14 and 18 with 3‐methyladenine (3‐MA), an inhibitor of autophagy. Immunoblot and immunofluorescence analysis demonstrated that autophagic mechanisms are dysregulated in the retina of OIR rats and indicated a possible correlation between autophagy and necroptosis, but not apoptosis. We found that 3‐MA acts predominantly by reducing autophagic and necroptotic markers in the OIR retinas, having no effects on apoptotic markers. However, 3‐MA does not ameliorate retinal function, which results compromised in this model. Taken together, these results revealed the crucial role of autophagy in retinal cells of OIR rats. Thus, inhibiting autophagy may be viewed as a putative strategy to counteract ROP.

## 1 INTRODUCTION

The retina is one of the highest oxygen demanding tissue in the body, and oxygen supply is guaranteed by a structured ocular vascular network.^1^ In humans, the retinal vascularization begins development during the fourth month of gestation and is completed before birth, at nine months. In the uterus, the hypoxic environment (pO_2_ of about 30 mmHg) allows retinal blood vessels to proliferate and migrate from the optic nerve to the retinal periphery, through vascular endothelial growth factor (VEGF)‐mediated angiogenesis.^2^ Infants born prematurely present an incompletely vascularized retina, characterized by a peripheral avascular zone, a hallmark of retinopathy of prematurity (ROP), a potentially blinding retinal vascular disease.^3^ ROP is constituted by two typical oxygen‐dependent phases. The first phase is induced by the hyperoxic extrauterine environment (pO_2_ of 150 mmHg) and triggered immediately after the premature birth; the increase in oxygen level initiates a decrease in VEGF, which causes the arresting of retinal vascular development. At this stage, the blood vessels are fragile and ineffective to reach the increasing demands of the developing retina, which becomes hypoxic. Consequently, the second phase starts with an increase in the levels of VEGF, which promotes an excessive growth of blood vessels that extend into the vitreous and, in worst cases, may lead to retinal detachment.^4^

In ROP, the retinal vasculature is not the only component of the retina to be damaged.^5^ Previous studies have demonstrated that multiple retinal cells in addition to endothelial cells are compromised.^6^ The rapid fluctuations in oxygen cause a substantial stress to the retina tissue, which can activate apoptotic pathways that lead to retinal cell death.^7^ In line with these findings, an expansion of studies suggests that autophagy is involved in retinal diseases and contributes to the progressive visual dysfunction.^8^, ^9^

Autophagy is an intracellular degradation system, essential to eliminate unnecessary cell components, including unfolded proteins and damaged organelles, through the intermediary formation of the autophagosome, a double membrane‐bound vesicle that fuses with the lysosome to promote the degradation of its content by the cellular lysosomal system.^10^ In healthy cells, autophagy is activated at low basal levels and has a crucial role to maintain cellular homeostasis, operating as a protective mechanism for adaptation to environmental stresses, including oxidative stress, hypoxia and nutritional starvation. At the molecular level, autophagy is regulated by several factors, which are involved in specific steps of the mechanism. As a consequence of nutrient limitation, the major positive regulator of autophagic machinery is the adenosine monophosphate‐activated protein kinase alpha (AMPKα), which phosphorylates the autophagic inducer, Unc‐51 like autophagy activating kinase (Ulk)1 on Ser^555^, promoting autophagy induction.^11^ By contrast, autophagy can be inhibited by the mammalian target of rapamycin (mTOR) kinase, which is phosphorylated and activated by protein kinase B (Akt). mTOR inhibits autophagy by phosphorylating Ulk1 on Ser^777^ site, and activates other translational regulators, including the initiation factor 4E‐binding protein (4E‐BP1), the ribosomal S6 protein kinase (S6K) and its substrate (S6), which have a crucial role to regulate the initiation of mRNA translation.^12^

However, in pathological conditions, an impairment of autophagy flux might be damaging and promote autophagic cell death, which is associated with an accumulation of autophagosomes in the cell.^13^ Thus, certain cell death mechanisms have been classified as ‘autophagy‐mediated’, when autophagy precedes and triggers the programmed cell death, which include necroptosis, a receptor‐interacting protein kinase (RIPK)‐dependent pathway, and apoptosis, a caspase‐dependent mechanism^14^, ^15^ Interestingly, several studies have suggested that the autophagy machinery might promote either apoptosis or necroptosis, based on the energetic state and ATP levels of the cells. Crucially, a high level of intracellular ATP promotes apoptosis, whereas a rapid drop in ATP is implicated in necrotic cell death.^16^, ^17^, ^18^, ^19^ In both cases, autophagy flux is impaired and an accumulation of autophagosomes may compromise cell viability.^20^, ^21^

In the present study, we used a rat model of oxygen‐induced retinopathy (OIR), an acknowledged model of ROP. In rats, the retinal vasculature develops postnatally, with a full development reached at around postnatal day (P) 13‐P16.^22^ In the rat model of OIR developed by Penn,^23^ the exposure of the newborn rats to alternating daily cycles of oxygen (50% and 10%, respectively) for the first 14 days delays the development of retinal vessels, causing the appearance of an avascular periphery. Returning to room air until P18 causes the growth of aberrant retinal vessels that leads to the formation of neovascular tufts.^24^ Neovascularization is further accompanied by a profound retinal dysfunction, all events seen in human ROP.^25^ In the present study, the rat model of ROP was used to evaluate changes in the expression of key mediators implicated in autophagy. In addition, a possible correlation between autophagy and programmed cell death pathways was examined by assessing the expression of autophagic, apoptotic and necroptotic markers in retinal endothelial cells and in several retinal layers of rat pups. In addition, an inhibitor of autophagy was evaluated to analyse its possible effects in mitigating autophagy‐associated cell death and retinal dysfunction in the OIR rat model.

## 2 MATERIAL AND METHODS

### 2.1 Animals

Rat pup litters were maintained with their own nursing mothers in a regulated environment (24 ± 1°C, 50 ± 5% humidity), with a 12 h light/dark cycle and provided with food and water *ad libitum*. Procedures involving animal experiments were performed in agreement with the Italian guidelines for animal care (DL 26/14) and the European Communities Council Directive (2010/63/UE). Experiment protocols were approved by the Ethical Committee in Animal Experiments of the University of Pisa.

### 2.2 Rat model of OIR and pharmacological treatments

Seventy‐two newborn rat pups with their nursing mothers were placed in an incubator and exposed to alternative daily hyperoxia and hypoxia cycles (respectively, 50% ± 2% and 10% ± 2% O_2_) for 14 days, and returned to room air (RA) until P18. Hyperoxia was obtained by increasing oxygen tension, while hypoxia by increasing nitrogen tension. Oxygen concentration was monitored with an oxygen sensor connected to the chamber (Pro‐Custom Elettronica). An equal number of rat pups were kept in RA as control.

Twenty‐four OIR rat pups were treated with intraperitoneal injection of 3 mg/kg of 3‐methyladenine (3‐MA; cat. no. sc‐205596; Santa Cruz Biotechnology) dissolved in normal saline solution. The treatment was performed daily from birth to P14 (12 rats) or P18 (12 rats). As controls, 24 OIR rat pups were injected with an equal volume of vehicle until P14 (12 rats) or P18 (12 rats). Twelve additional OIR rat pups treated with the lysosomal inhibitor chloroquine (CQ) were also used (see Supporting information). Retinas were collected at P14 and P18 from both males and females, as there was no apparent gender difference. Rat pups were anaesthetized with an intraperitoneal injection of 30 mg/kg of pentobarbital, and the animals were euthanized by cervical dislocation.

### 2.3 Whole‐retina vascular area

A total of 24 rat pups at P14 and P18 exposed to RA or OIR (six rats for each time point per oxygenation paradigm) were prepared as previously described.^26^ Briefly, free‐floating retinal whole‐mounts were stained with fluorescein‐labelled isolectin B4 (1:200; Vector Laboratories). Images were acquired by fluorescence microscopy (Ni‐E; Nikon‐Europe), and the percentual vascular area was determined with the ImageJ freeware.

### 2.4 Western blot analysis

Retinas, from both OIR and RA rat pups, were lysed with RIPA lysis buffer (Millipore Corporation), containing phosphatase inhibitor (PhosStop, Roche) and protease inhibitor (Sigma‐Aldrich) cocktails. Protein concentration was determined using a micro‐BCA method (Thermo Fisher Scientific), and 15 µg of total proteins was separated by SDS‐PAGE and transferred to polyvinylidene difluoride (PVDF) membranes (Millipore Corporation).

PVDF membranes were blocked at room temperature (RT) for 1 h, with 5% skimmed milk, except for membranes used to detect phosphorylated proteins, which were blocked with 4% bovine serum albumin (Sigma‐Aldrich), both dissolved in Tris‐buffered saline (TBS; Bio‐Rad Laboratories). Membranes were incubated overnight (ON) at 4°C with primary antibodies (Table 1). Secondary antibodies anti‐rabbit‐IgG and anti‐mouse‐IgG conjugated to horseradish peroxidase (1:10,000; cat. no. P044801‐2 and P016102‐2, respectively; Dako) were incubated for 1 h at RT. Finally, protein bands were visualized using the Clarity Western ECL substrate with a ChemiDoc XP imaging system (Bio‐Rad Laboratories). Densitometric analysis was performed by normalizing the target‐related optical density (OD) to the OD of either β‐actin or total non‐phosphorylated protein as appropriate.

**TABLE 1:** List of primary antibodies

Antibody	Dilution ratio	Company; Cat. No
Rabbit polyclonal anti‐p‐Ulk1 (Ser^757^) Rabbit monoclonal anti‐p‐Ulk1 (Ser^555^)	1:500 (WB) 1:500 (WB)	Cell Signaling Technology; 6888 Cell Signaling Tech.; 5869
Rabbit monoclonal anti‐Ulk1	1:1,000 (WB)	Cell Signaling Tech.; 8054
Rabbit monoclonal anti‐p‐AMPKα (Thr^172^)	1:500 (WB)	Cell Signaling Tech; 2535
Rabbit monoclonal anti‐AMPKα	1:1,000 (WB)	Cell Signaling Tech; 5832
Rabbit polyclonal anti‐ SQSTM1/p62	1:500 (WB) 1:100 (IF)	Abcam; ab91526
Rabbit polyclonal anti‐LC3	1:1,000 (WB) 1:100 (IF)	Cell Signaling Tech.; 4108
Rabbit monoclonal anti‐p−4E‐BP1 (Thr^37/46^)	1:500 (WB)	Cell Signaling Tech.; 2855
Rabbit monoclonal anti−4E‐BP1	1:500 (WB)	Cell Signaling Tech.; 9644
Rabbit monoclonal anti‐β‐actin	1:5,000 (WB)	Sigma‐Aldr.; SAB5600204
Rabbit polyclonal anti‐p‐Akt (Ser^473^)	1:500 (WB)	Cell Signaling Tech.; 9271
Rabbit polyclonal anti‐Akt	1:1,000 (WB)	Cell Signaling Tech.; 9272
Rabbit polyclonal anti‐p‐p70 S6 Kinase (Thr^389^)	1:1,000 (WB)	Cell Signaling Tech.; 9205
Rabbit monoclonal anti‐p70 S6 Kinase	1:500 (WB)	Cell Signaling Tech.; 2708
Rabbit polyclonal anti‐p‐S6 (Ser^240/244^)	1:500 (WB)	Cell Signaling Tech.; 2215
Mouse monoclonal anti‐S6	1:1,000 (WB)	Cell Signaling Tech.; 2317
Rabbit polyclonal anti‐active caspase−8	1:1,000 (WB) 1:200 (IF)	Novus Biol.; 56116
Rabbit polyclonal anti‐active caspase−3	1:500 (WB) 1:100 (IF)	Abcam; ab2302
Rabbit polyclonal anti‐RIPK−1	1:500 (WB) 1:100 (IF)	Novus Biol.; 77077
Rabbit polyclonal anti‐RIPK3	1:500 (WB) 1:200(IF)	Novus Biol.; 77299
Isolectin B4‐biotin	1:1,000 (IF)	Thermo Fisher; I21414

Abbreviations: IF, immunofluorescence; WB, Western blot.

### 2.5 Tissue preparation for immunofluorescence

Both OIR and RA eyes, untreated or treated with 3‐MA, were collected and used for retina sections. Eyeballs were fixed in 4% paraformaldehyde in 0.1 M phosphate buffer, pH 7.4, at RT for 24 h before processed for paraffin embedding. Four micrometre sections were subsequently deparaffinized in xylene. After rehydration into TBS, retinal sections were kept for 30 min in 10 mM citrate buffer (pH 6.0; Sigma‐Aldrich) with Tween‐20 (1:2,000; Sigma‐Aldrich) at 96°C. After rinsing in TBS, retina sections were incubated in a humidified chamber with 10% normal donkey serum (Abcam) diluted in TBS containing 5% (w/v) IgG and protease‐free bovine serum albumin (Jackson Immunoresearch) for 30 min. Primary antibodies (Table 1) were incubated ON at 4°C, while secondary antibodies, anti‐rat‐Alexa 546 (1:500; cat. no. A11081; Sigma‐Aldrich) and anti‐goat‐Alexa 647 (1:500; cat. no. SAB4600175; Sigma‐Aldrich) were incubated for 1 h at RT. Sections were mounted with Vectashield mounting with DAPI, and images were acquired on an Axioskop 2 plus fluorescence microscope with the AxioVision software (Zeiss).

### 2.6 ERG recording

Retinal function was examined with scotopic full‐field ERG recorded from P18 rat pups. Before ERG testing, rats were dark adapted for a minimum of 16 h and their manipulation was done under dim red light. Briefly, both RA and OIR rat pups, untreated or treated with 3‐MA, were anaesthetized by intraperitoneal injection of pentobarbital (30 mg/kg); pupils were dilated with 0.5% atropine, and a heating pad was used to keep the body temperature at 38 °C. The electrophysiological signals were recorded through silver/silver chloride ring electrodes inserted under the lower eyelids. The cornea was intermittently irrigated with saline solution to prevent clouding of the ocular media. Electrodes in each eye were referred to a needle electrode inserted subcutaneously at the level of the corresponding frontal region. The ground electrode was placed on the tail. Mixed (rod and cone) responses were evoked by flashes of different light intensities ranging from −1.5 to 1 log cd‐s/m^2^ generated through a Ganzfeld stimulator (Biomedica Mangoni), averaging 5 different ERG responses obtained with an interval of 20 seconds between light flashes. The electrodes were connected to a two‐channel amplifier. Signals were amplified at 1,000 gain and bandpass filtered between 0.2 and 500 Hz before being digitized at 5 kHz rate with a data acquisition device (Biomedica Mangoni). The ERG responses were first analysed to evaluate the amplitude of a‐ and b‐waves, and the data were graphed to determine any gross changes in the intensity‐response function for that eye. The amplitude of the a‐wave was measured at a fixed time of 8 ms after stimulus onset to minimize contamination from non‐photoreceptoral cell contributions.^27^ The b‐wave amplitude was measured from the trough of the a‐wave to the peak of the b‐wave or, if no a‐wave was present, from the prestimulus baseline. Intensity‐response functions of the b‐wave were fit to a modified Naka‐Rushton function^28^ V(I) = V0 + (Vmax In)/ (In +kn). In this equation, V is the amplitude of the b‐wave (in μV), I is the stimulus intensity (in log cd‐s/m^2^), V0 is the non‐zero baseline effect, Vmax is the saturated amplitude of the b‐wave (in μV), k is the stimulus intensity that evokes a b‐wave of half‐maximum amplitude (in log cd‐s/m^2^), and n, which was constrained to unity, is a dimensionless constant controlling the slope of the function and represents the degree of heterogeneity of retinal sensitivity. Mean amplitudes of ERG responses were plotted as a function of increasing light intensities. For each experimental condition, ERG analysis was performed on 6 rats for each group.

### 2.7 Statistical analysis

Statistical analysis was performed applying one‐way or two‐way ANOVA, as appropriate, followed by Bonferroni's multiple comparison post‐test using the GraphPad Prism software. The results are expressed as mean ± standard error of the mean (SEM) of *n* = 6. Differences with *p* < .05 were considered statistically significant.

## 3 RESULTS

### 3.1 Increased VEGF modulates retinal vasculature in OIR rats

**FIGURE 1:**
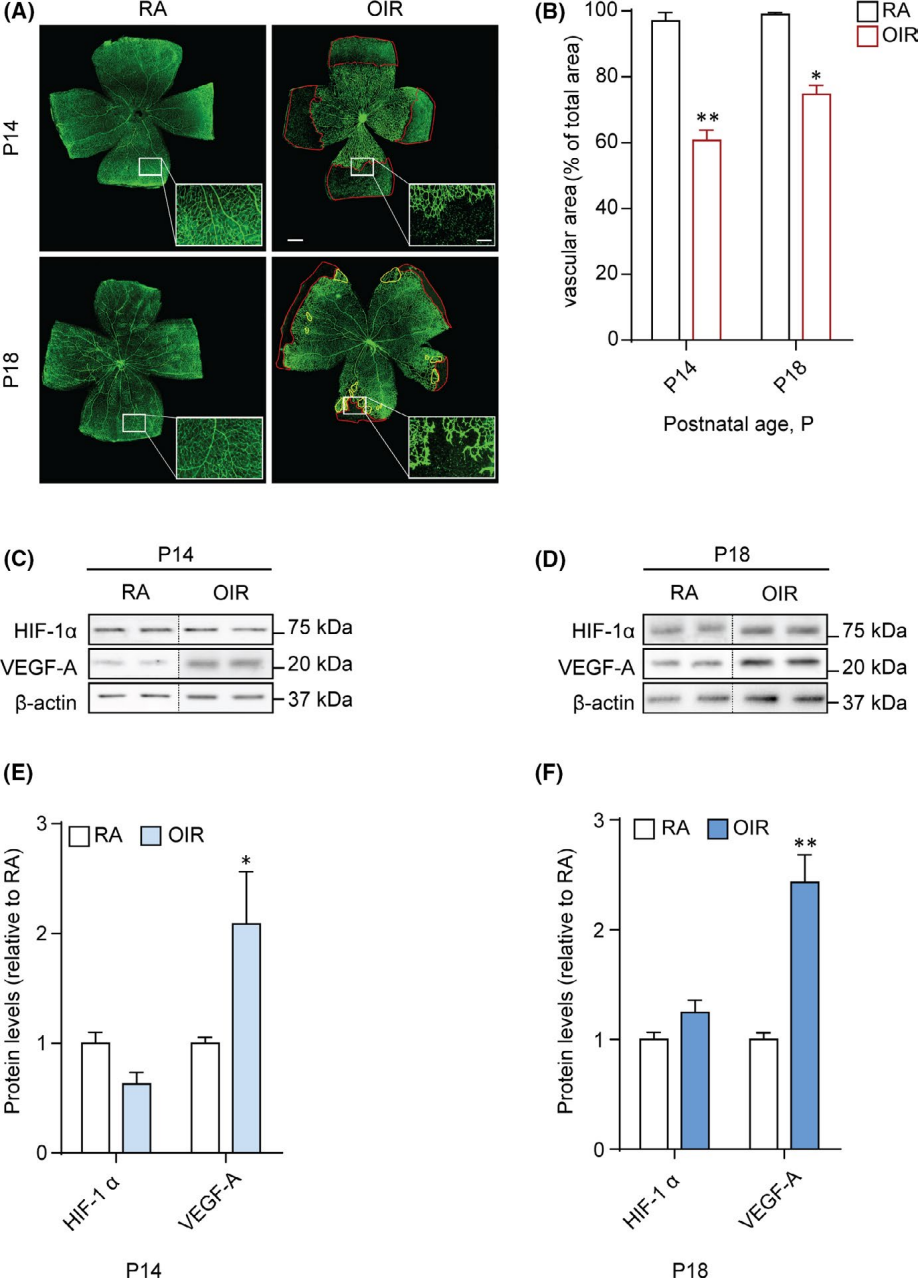
Vascular network and angiogenic markers in OIR rat pups. (A) Visualization of blood vessels by isolectin B4 staining of both RA and OIR rat pup retinas, at P14 and P18. The avascular (red boundaries) and the tuft (yellow boundaries) areas are shown. Scale bar =500 µm (100 µm in the insets). (B) Vascular area of the retina presented as % of total area. (C, D) Representative immunoblots of HIF‐1α, VEGF‐A and β‐actin (loading control) of rat pups exposed to RA or OIR at P14 and P18. (E, F) Densitometric analysis of the immunoreactive bands at P14 (E) and P18 (F). Data are plotted as mean ± SEM. Differences between groups were tested for statistical significance using one‐way ANOVA followed by Bonferroni's multiple comparisons post‐test (n=6 animals per group). *p < .05 and **p < .01 vs respective P14 and P18 RA

The exposure of newborn rats to the OIR protocol delays the development of retinal vessels, causing peripheral retinal avascularity at P14, and at P18 the formation of neovascular tufts, as a consequence of the aberrant retinal vessels' growth.^24^ Here, this vascular phenotype was confirmed by whole‐retina vasculature analysis using isolectin B4 (Figure 1a), to visualize the retinal blood vessels’ area (Figure 1b).

In ROP, low oxygen levels play a crucial role in the development of the hypoxic phase of pathogenesis, through the transcription of hypoxia‐induced factor (HIF)‐1–dependent VEGF.^23^, ^29^ As such, we assessed a Western blot analysis to monitor protein levels of both HIF‐1α (the oxygen‐dependent subunit of HIF‐1) and VEGF‐A in the retina on P14 and P18 (Figure 1c,d). Densitometric analysis showed no significant difference in HIF‐1α levels between OIR and RA‐exposed rats on both P14 and P18. However, on P14, OIR retinas showed a significant increase in VEGF‐A level (*p *< 0.05), which further increased at P18 (*p *< 0.01 vs RA) (Figure 1e,f).

### 3.2 Autophagic flux is altered in the OIR retina

**FIGURE 2:**
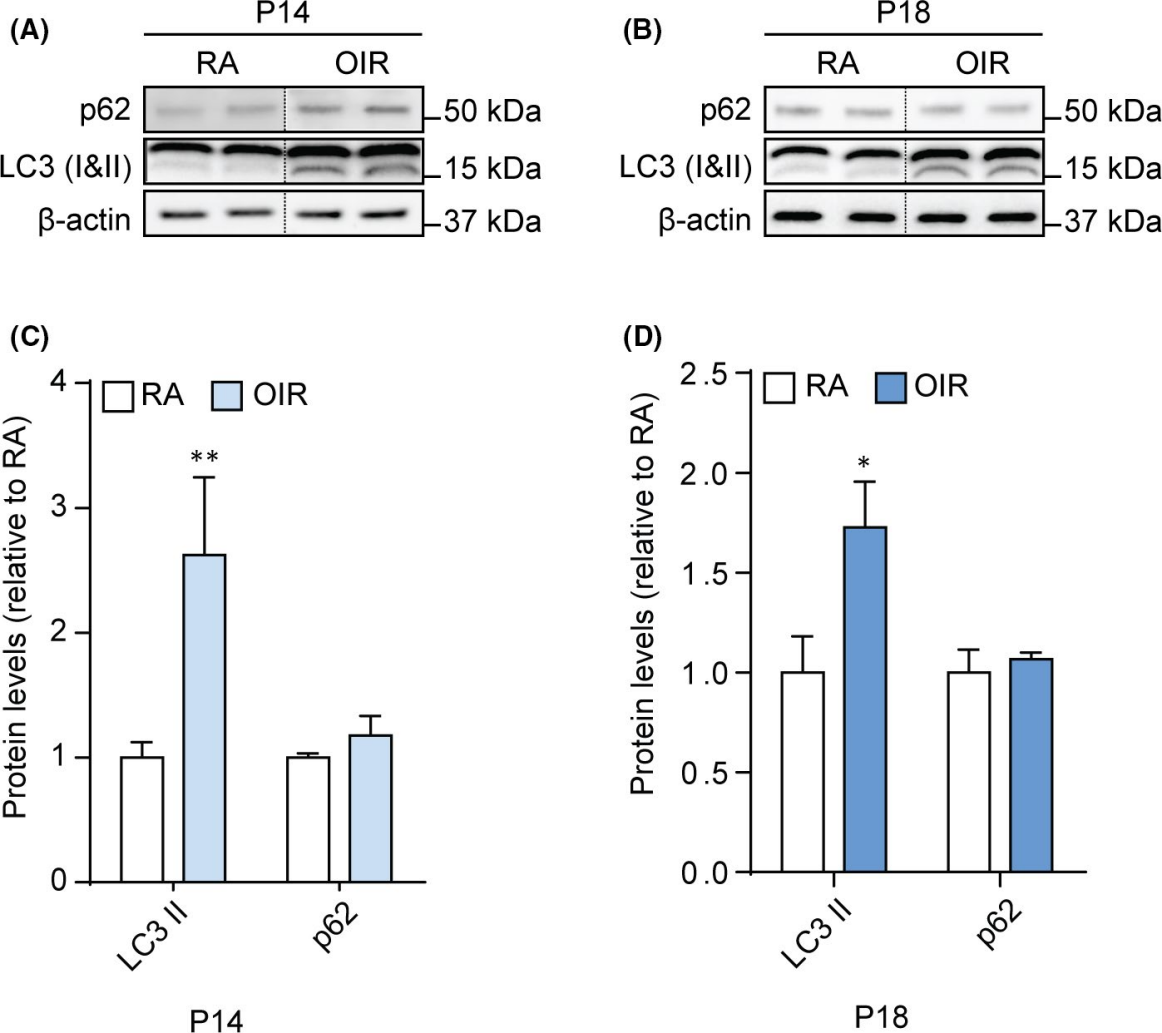
Levels of autophagic markers in retinas of RA and OIR rat pups. (A, B) Representative immunoblots of p62, LC3‐I, LC3‐II and β‐actin (loading control) in RA and OIR retinas at P14 and P18. (C, D) Densitometric analysis of the immunoreactive bands at P14 (C) and P18 (D). Data are plotted as mean ± SEM. Differences between groups were tested for statistical significance using one‐way ANOVA followed by Bonferroni's multiple comparisons post‐test (n=6 animals per group). *p < .01 and **p < .001 vs respective P14 and P18 RA

To evaluate a putative variation in autophagic markers, we performed a Western blot analysis on protein extracts from both RA and OIR retinas on P14 and P18 (Figure 2a,b). We analysed protein levels of microtubule‐associated protein 1‐light chain 3 (LC3) and sequestosome 1 (p62), acknowledged markers involved in autophagic mechanisms.^30^ LC3 exists as a cytosolic protein, LC3‐I, which can be lipidated to form LC3‐phosphatidylethanolamine conjugate (LC3‐II) that is recruited to autophagosomal membranes,^31^ while p62 is a cargo for degradation of ubiquitinated proteins via autophagy that decreases in presence of activated autophagy.^32^ Densitometric analysis demonstrated a significant increase of LC3‐II in OIR retinas on P14, when compared to RA (*p* < .001). A similar response of LC3‐II was observed on P18 (*p* < .01). No significant difference in p62 levels was observed on both P14 and P18 (Figure 2c,d). To evaluate the autophagic flux, we assessed LC3‐II and p62 levels in OIR rats treated with 3‐MA or CQ (Figure S1). While 3‐MA prevented the OIR‐induced increase in LC3‐II at both P14 and P18, CQ did not affect LC3‐II levels. The levels of p62 in the OIR retina were not affected by either 3‐MA or CQ.

### 3.3 OIR modulates autophagy by inactivating the Akt‐mTOR anti‐autophagic pathway, while activating the AMPKα pro‐autophagic pathway

**FIGURE 3:**
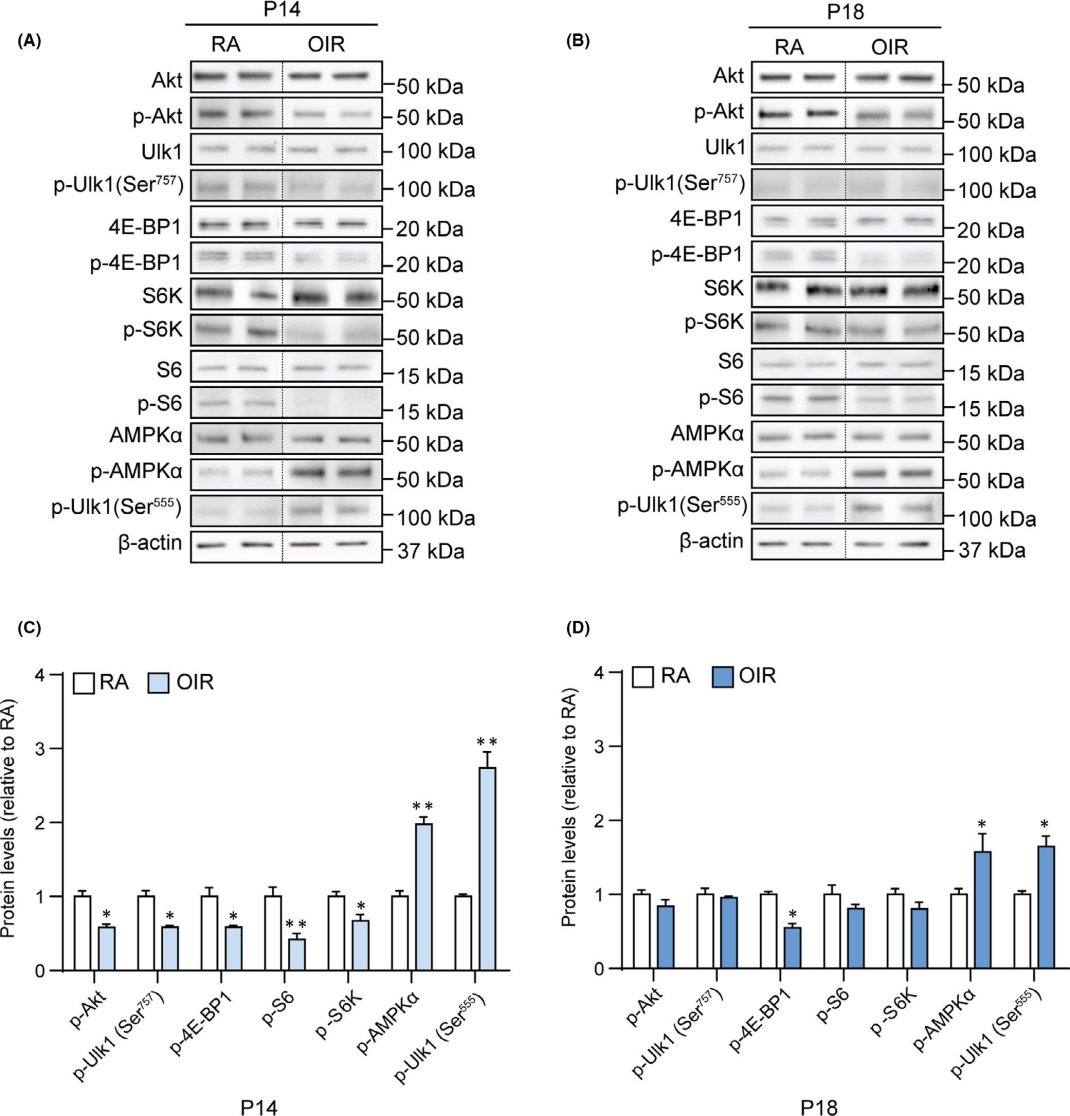
Levels of anti‐ and pro‐autophagic molecules in retinas of RA and OIR rat pups. (A, B) Representative immunoblots of Akt, Ulk1, 4E‐BP1, S6K, S6, AMPKα, their phosphorylated forms and β‐actin (loading control) in the retina of RA and OIR rats at P14 and P18. (C, D) Densitometric analysis of phosphorylated protein levels normalized to the respective total protein at P14 (C) and P18 (D). Data are plotted as mean ± SEM. Differences between groups were tested for statistical significance using one‐way ANOVA followed by Bonferroni's multiple comparisons post‐test (n=6 animals per group). *p < .05 and **p < .01 vs respective P14 and P18 RA

To evaluate the role of the Akt‐mTOR and the AMPKα pathways in the OIR‐induced activation of early autophagic stages, Western blot analysis was performed to analyse the phosphorylation status of both anti‐autophagic molecules Akt, Ulk1 (phosphorylated at Ser^757^), 4E‐BP1, S6K and S6, and pro‐autophagic molecules AMPKα and Ulk1 (phosphorylated at Ser^555^) (Figure 3a,b). Densitometric analysis demonstrated that in OIR, p‐Akt, p‐Ulk1 (Ser^757^), p‐4E‐BP1, p‐S6K (*p* < .05) and p‐S6 (*p *< .01) were decreased at P14. In contrast, both p‐AMPKα and p‐Ulk1 (Ser^555^) were increased at the same time point (*p* < .01) (Figure 3c). At P18, in the OIR retinas, we observed a decrease in p‐4E‐BP1 (*p* < .05), with no statistical difference in p‐Akt, p‐Ulk1 (Ser^757^), p‐S6K and p‐S6 levels, concomitant with an increase in the levels of both p‐AMPKα and p‐Ulk1 (Ser^555^) (*p* < .05) (Figure 3d).

### 3.4 3‐methyladenine reduces autophagic and necroptotic markers in the OIR retina

**FIGURE 4:**
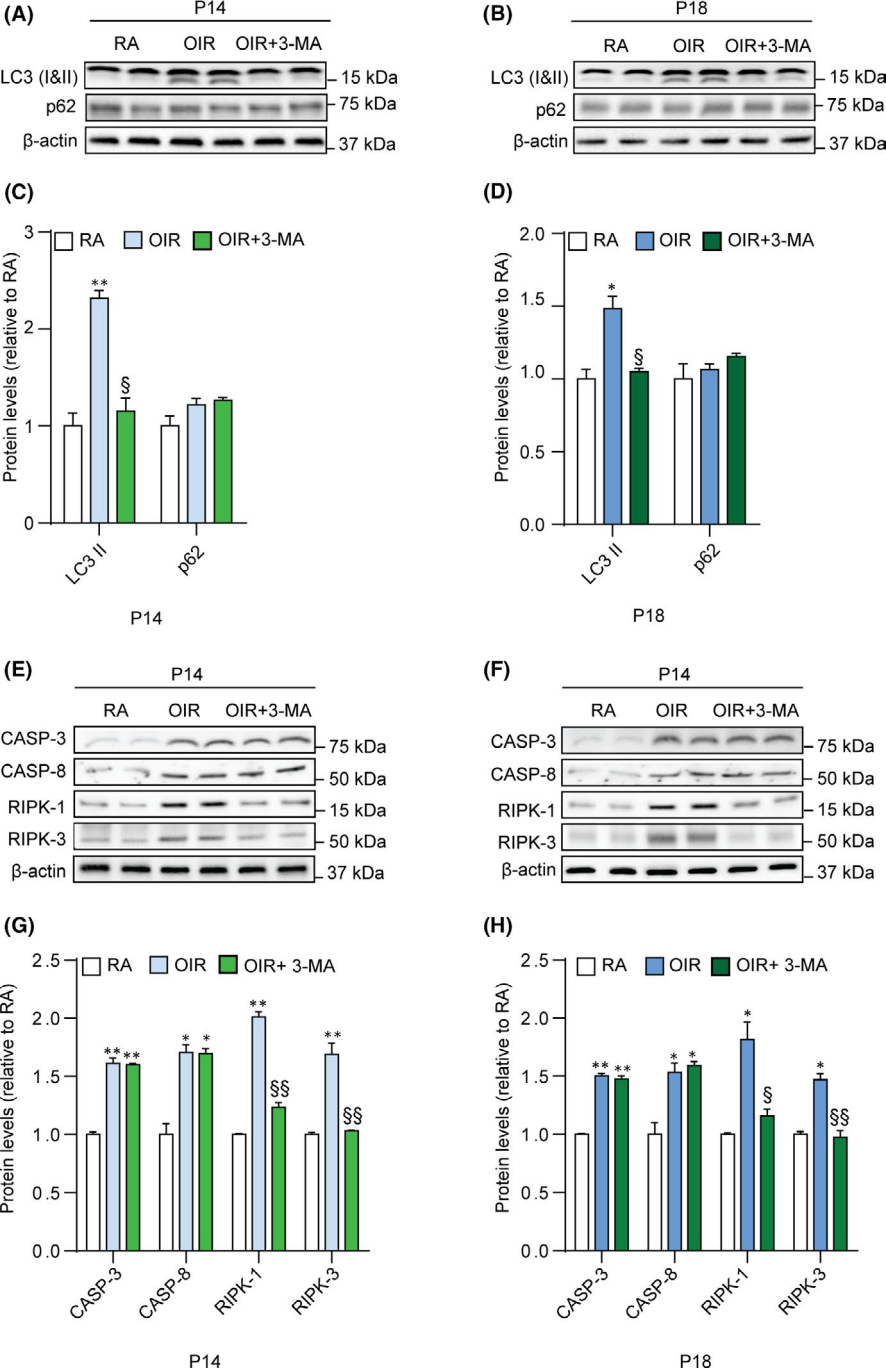
Levels of autophagic, apoptotic and necroptotic markers in RA, OIR and OIR +3‐MA rats. (A, B) Representative blots of LC3‐I, LC3‐II, p62 and β‐actin (loading control) in RA, OIR and OIR +3‐MA rats at P14 and P18. (C, D) Densitometric analysis of the immunoreactive bands at P14 (C) and P18 (D). (E, F) Representative blots of active caspase 3 (Casp‐3), active caspase 8 (Casp‐8), RIPK‐1, RIPK‐3 and β‐actin (loading control) in RA, OIR and OIR +3‐MA rats at P14 (E) and P18 (F). (G, H) Densitometric analysis of the immunoreactive bands at P14 (G) and P18 (H). Data are plotted as mean ± SEM. Differences between groups were tested for statistical significance using one‐way ANOVA followed by Bonferroni's multiple comparisons post‐test (n=6 animals per group). *p < .05 and **p < .01 vs respective P14 and P18 RA; ^§^ *p* < .05 and ^§§^ *p* < .01 vs respective P14 and P18 OIR

**FIGURE 5:**
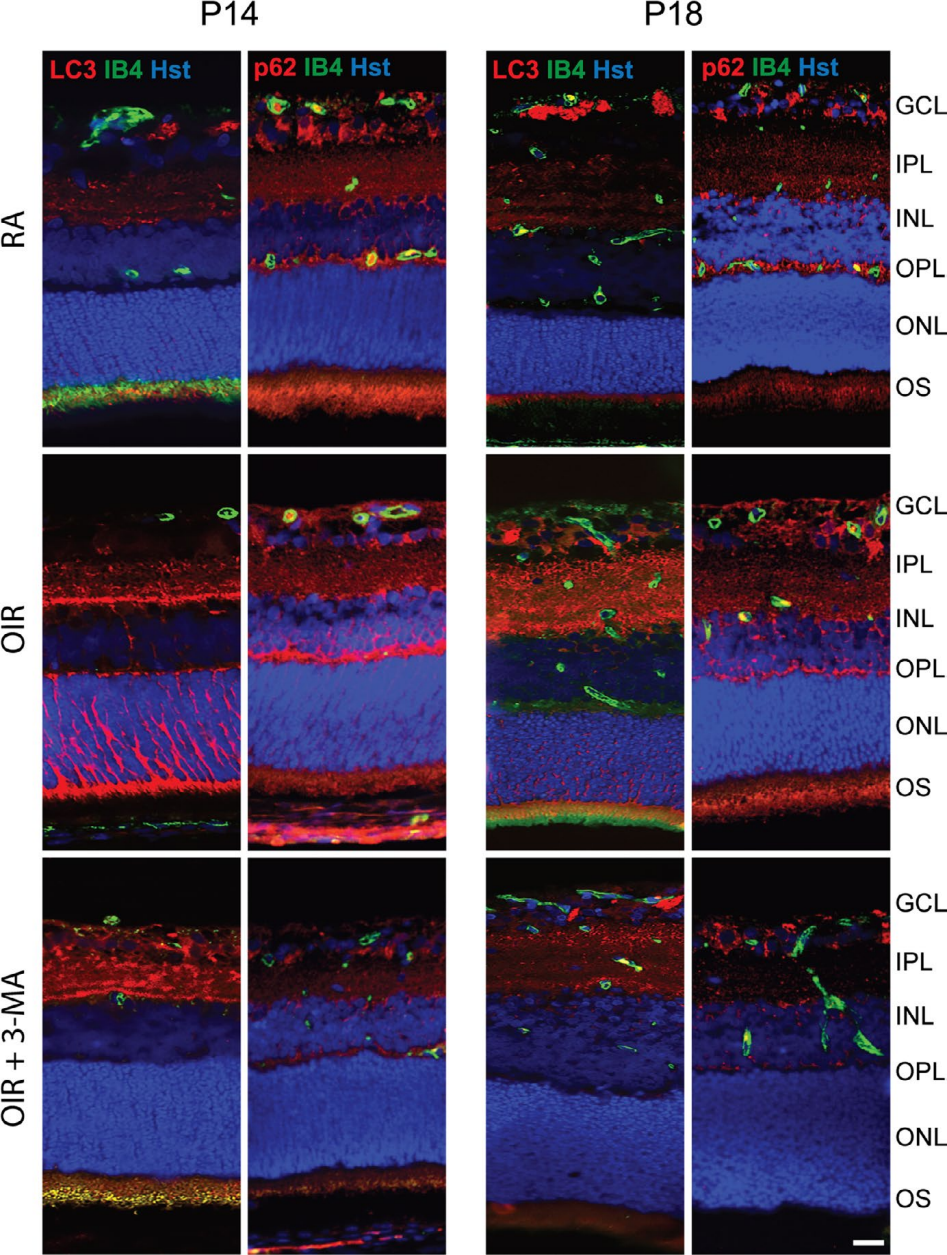
Representative immunofluorescence images of LC3 and p62 (red), isolectin B4 (IB4; green) and Hoechst (Hst; blue) in retina sections of RA, OIR and OIR +3‐MA rats at P14 and P18. GCL, ganglion cell layer; IPL, inner plexiform layer; INL, inner nuclear layer; OPL, outer plexiform layer; ONL, outer nuclear layer; and OS, outer segments of photoreceptors (*n* = 6 sections per group). Scale bar = 50 µm

**FIGURE 6:**
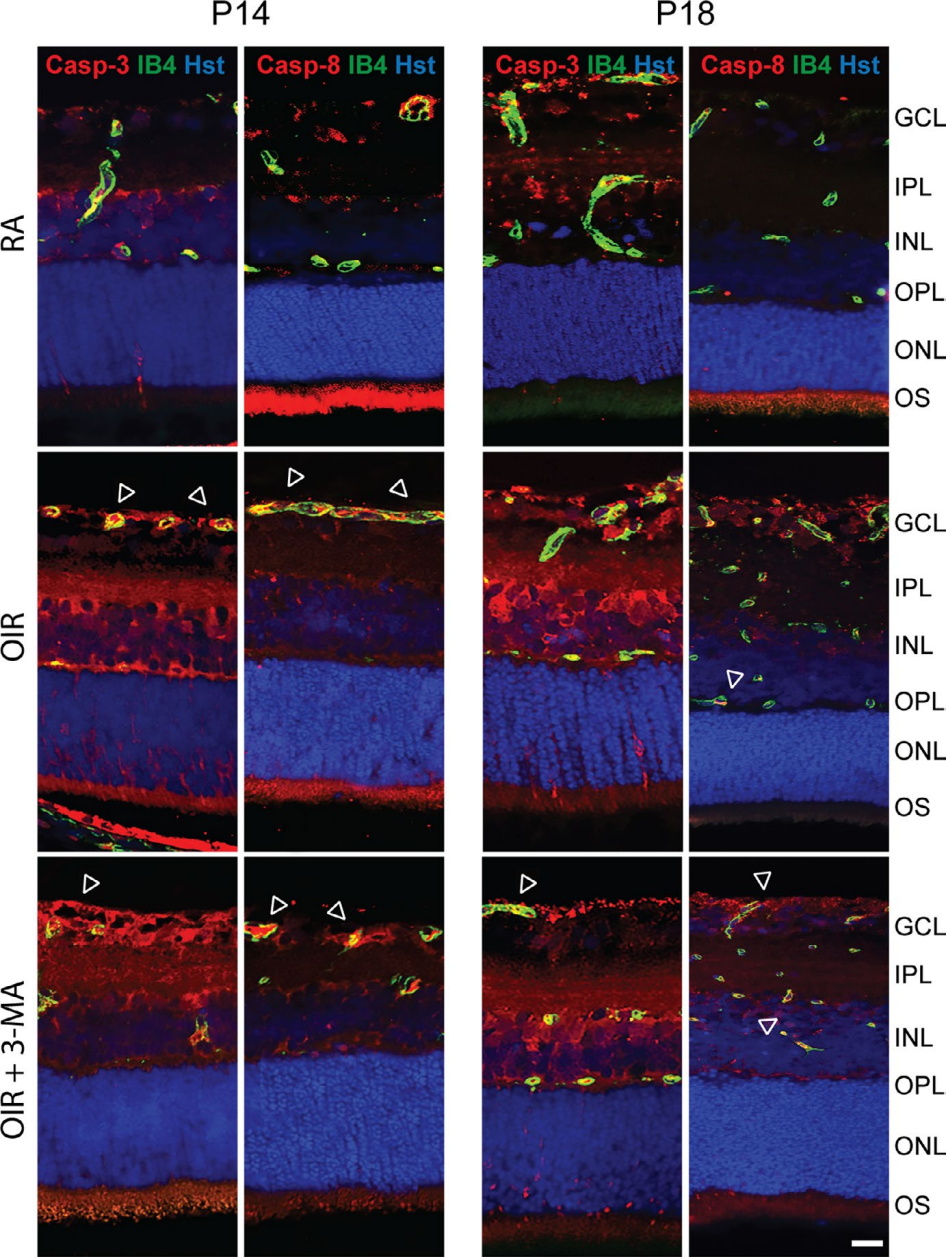
Representative immunofluorescence images of active caspase 3 and active caspase 8 (Casp‐3 and Casp‐8, respectively; red), isolectin B4 (IB4; green) and Hoechst (Hst; blue) in retina sections of RA, OIR and OIR +3‐MA rats at P14 and P18. Arrowheads indicate colocalization of either active caspase 3 or active caspase 8 with IB4. GCL, ganglion cell layer; IPL, inner plexiform layer; INL, inner nuclear layer; OPL, outer plexiform layer; ONL, outer nuclear layer; and OS, outer segments of photoreceptors (*n* = 6 sections per group). Scale bar =50 µm

**FIGURE 7:**
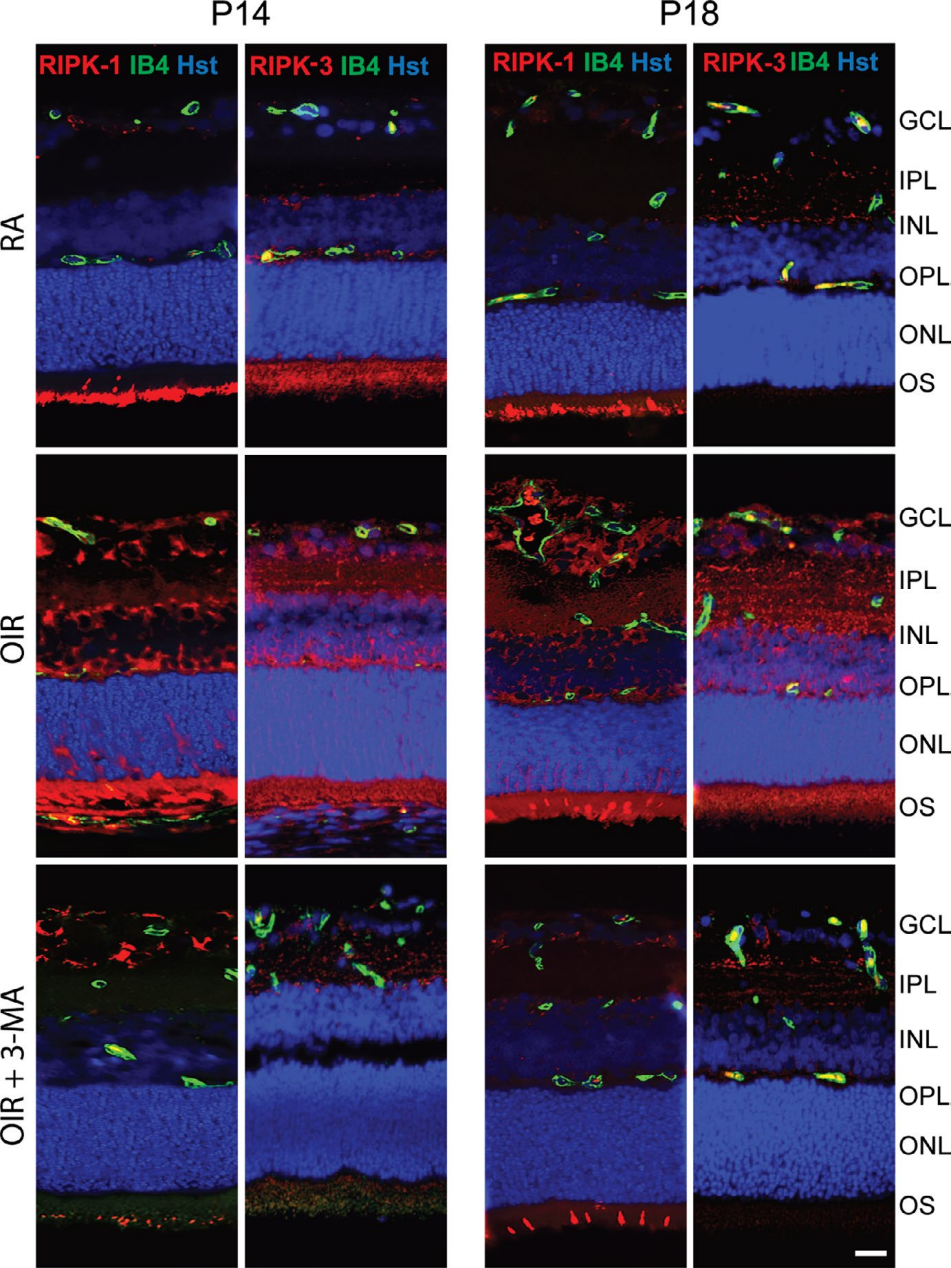
Representative immunofluorescence images of RIPK‐1 and RIPK‐3 (red), isolectin B4 (IB4; green) and Hoechst (Hst; blue) in retina sections of RA, OIR and OIR +3‐MA rats at P14 and P18. GCL, ganglion cell layer; IPL, inner plexiform layer; INL, inner nuclear layer; OPL, outer plexiform layer; ONL, outer nuclear layer; and OS, outer segments of photoreceptors (n =6 sections per group). Scale bar = 50 µm

To validate the efficacy of 3‐MA treatment in preventing the activation of autophagy, necroptosis and apoptosis, we performed Western blot analysis for markers of these three pathways, both at P14 and P18. The treatment with 3‐MA significantly inhibited the OIR‐induced upregulation in LC3‐II levels at P14 and P18 (*p* < .05), while not influencing p62 levels (Figure 4a‐d). In addition, OIR induced an increase in active caspase 3 (*p* < .01) and caspase 8 (*p* < .05) levels at both P14 and P18, which was unaffected by 3‐MA (Figure 4e‐h). Moreover, OIR retinas showed an increase in RIPK‐1 and RIPK‐3 levels at both P14 and P18 (*p* < .01 at P14 and *p* < .05 vs P18), which was prevented by 3‐MA (Figure 4e‐h). To further visualize the variation of autophagy into the retina, immunostained retina sections of RA, OIR and 3‐MA‐treated OIR rats were assessed at both P14 and P18, using LC3 and p62 as autophagic markers and isolectin B4 as an endothelial cell marker. In addition, to clarify a possible relationship between autophagy and mechanisms of cell death, the sections were also stained for active caspase 3 and active caspase 8 as apoptotic markers and for RIPK‐1 and RIPK‐3 as necroptotic markers. Immunofluorescence signals demonstrated an overall variation of autophagic (Figure 5), apoptotic (Figure 6) and necroptotic (Figure 7) markers in the OIR retinas. In particular, a predominant expression of LC3, RIPK‐1 and RIPK‐3 was visualized in the ganglion cell layer (GCL), the inner plexiform layer (IPL), the outer plexiform layer (OPL), the outer nuclear layer (ONL) and the outer segment (OS), while a colocalized expression of active caspase 3, active caspase 8 and isolectin B4 was observed in OIR retinas, mainly in the GCL. On the contrary, p62 expression, which was mainly localized in the IPL, the OS and the ONL, was not affected by the OIR protocol. As depicted in Figures 3, 5, 6, 7, 3‐ MA treatment reduced the expression of LC3, RIPK‐1 and RIPK‐3 on both P14 and P18; in contrast, no detectable reduction of p62, as well as of active caspase 3 and active caspase 8, was observed after 3‐MA. In light of the significant increase of active caspase 3 and active caspase 8 in OIR rats, we evaluated DNA fragmentation. A TUNEL staining was performed on retina sections from RA, OIR and 3‐MA‐treated OIR rats at P14 and P18 (Figure S2). However, we did not observe any TUNEL‐positive signal in the studied retinas, suggesting that nuclear independent cell death mechanisms would be predominant in our study.

### 3.5 3‐Methyladenine does not affect retinal function

As previously described, newborn rats exposed to the OIR protocol are characterized by visual dysfunction.^33^ By recording ERG, we measured retinal function at P18 in order to determine whether the effects of 3‐MA on autophagy were accompanied by recovered visual function.

**FIGURE 8:**
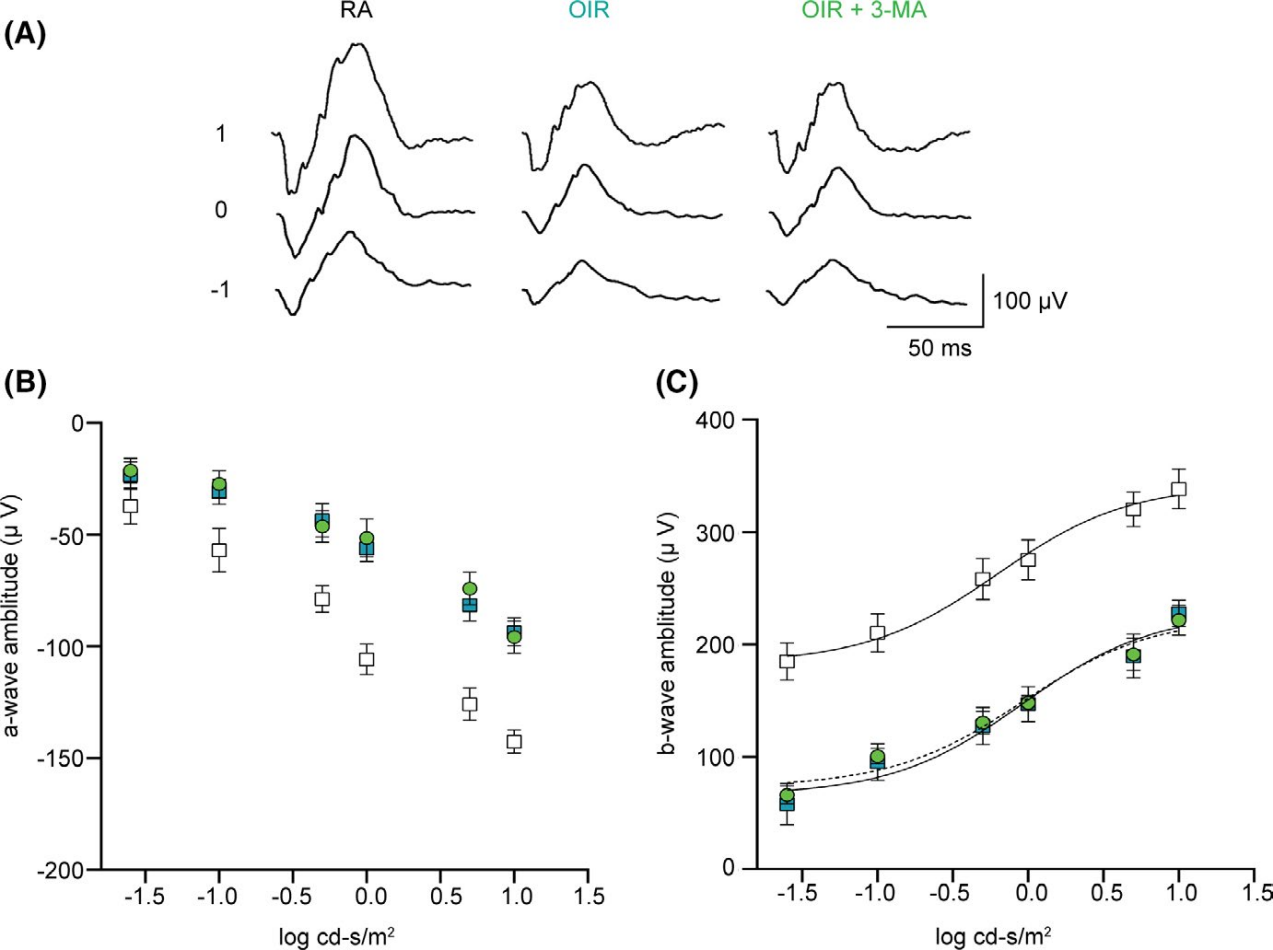
Retinal function in RA, OIR and OIR +3‐MA rats. (A) Representative ERG waveforms recorded at a light intensity of −1, 0 and 1 log cd‐s/m.^2^ (B, C) Scotopic a‐wave (B) and b‐wave amplitudes (C) plotted as a function of increasing light intensity in RA (white squares and black line) and in OIR rats untreated (blue squares and black line) or treated with 3‐MA (green circles and dotted line). Data are plotted as mean ± SEM. Differences between groups were tested for statistical significance using two‐way ANOVA followed by Bonferroni's multiple comparisons post‐test (*n* = 6 animals per group)

Representative mixed a‐ and b‐waves recorded from RA and OIR rats either untreated or 3‐MA‐treated are shown in Figure 8A. Increased a‐ and b‐wave amplitudes with increasing stimulus intensity were observed (Figure 8B,C). As expected, in response to hypoxia, rats displayed reduced a‐wave amplitude (*p *< 0.001 from −1.0 to 1 log cd‐s/m^2^) and b‐wave amplitude (*p* < .001 from −1.5 to 1 log cd‐s/m^2^). Similar results were obtained in OIR rats treated with 3‐MA. The amplitudes of b‐waves obtained over varying flashlight intensities were fitted using the Naka‐Rushton equation to evaluate the post‐receptor response amplitude (Vmax) and the retinal sensitivity (k). ^27^, ^28^ In OIR rats, both Vmax and k were significantly lower than in RA rats, with Vmax of 229.1 ± 18.5 µV with respect to 342.3 ± 16.5 µV (*p* < .001) and k of −0.03 ± 0.15 log cd‐s/m^2^ with respect to −0.20 ± 0.12 log cd‐s/m^2^ (*p* < .05). After 3‐MA treatment, the values of Vmax and k were not significantly different from those in untreated OIR rats, with Vmax of 225.0 ± 11.5 µV and k of −0.03 ± 0.13 log cd‐s/m^2^.

## 4 DISCUSSION

ROP is a vascular disorder, characterized by abnormal proliferation of retinal blood vessels.^3^ Simultaneously, ROP is often accompanied by other events, such as inflammation and neurodegeneration, which might further compromise the visual function of preterm babies.^34^ Increasing evidence has suggested that autophagic mechanisms of cell death are involved in ocular diseases.^35^ Canonically, autophagy operates as a cytoprotective process, degrading toxic organelles or proteins through a recycle system. Albeit, in stress conditions, prolonged activation of autophagy contributes to the execution of programmed cell death, which is closely associated with the development of diseases.^36^Due to its high metabolic demand, the retina is characterized by a strong expression of autophagy proteins, and their imbalance might compromise the function of the neuroretina.^8^ In a rat model of retinal ischaemia and in an *in vivo* model of glaucoma, a prolonged activation of autophagy promotes cell death in retinal neurons, and its inhibition through 3‐MA suppresses autophagosome formation and reduces cell death.^37^, ^38^ On the contrary, in an *ex vivo* model of diabetic retinopathy and in a mouse model of OIR, an induction of autophagy mediates neuroprotection, by reducing cell death events in retinal cells.^39^, ^40^ Thus, autophagic mechanism, if not balanced, could represent a double‐edged sword in retinal cells.

In the present study, we investigated the involvement of autophagy in a rat model of OIR, and we found evidence that autophagy is activated in several retinal layers of OIR rats. In addition, we found that excessive autophagy could involve the activation of necroptotic mechanisms, leading to retinal cell death. In conformity, we observed a reduction of retinal cell death by using the inhibitor of autophagy, 3‐MA.

Initially, before exploring changes in the autophagy flux in the retina of OIR rats, we monitored protein levels of HIF‐1α and VEGF‐A, the main factors involved in neovascularization both in the OIR animal models and in ROP‐afflicted babies.^4^ In the rat model, a previous study has reported a significant increase in VEGF levels in pup rats exposed to the OIR protocol, when compared with rats kept in RA.^41^ This is in agreement with our data showing a peak of VEGF in OIR rats at both P14 and P18. As demonstrated, the increase of VEGF‐A levels is due to an adaption to supplements deficiency, which can be developed by the hypoxic stimulus from retinal cells.^42^ In fact, as a consequence of the oxygen fluctuations induced in this model, the retinal blood vessels are fragile, structurally deficient and poorly organized; thus, they are not able to fully nourish oxygen and nutrients to retinal cells.^43^ Nevertheless, no significant difference in HIF‐1α levels has been detected in OIR retinas at P14 and P18, suggesting that in this model the VEGF‐mediated angiogenesis might be induced through HIF‐1–independent signalling pathways, as previously demonstrated in human retinal pigment epithelial cells.^44^, ^45^ The possibility exists that at least one of them may involve AMPKα, which has been reported to contribute to VEGF upregulation in endothelial cells.^46^, ^47^ It is indeed putative that the oxygen fluctuations in the rat OIR model could result in a low‐grade, HIF‐1–independent cellular hypoxia with elevated intracellular reactive oxygen species^48^, ^49^ that lead to starvation signals and AMPKα‐mediated VEGF‐A expression.

In addition, energy deprivation‐promoted AMPKα activation induces autophagic mechanisms by inhibition of mTOR and a direct phosphorylation of autophagy modulators.^11^ Interestingly, here in OIR rats we observed an increase of p‐AMPKα and p‐Ulk1 (Ser^555^) protein levels, with a significant upregulation of LC3‐II on both P14 and P18 retinas. Concomitantly, a critical reduction was observed on Akt‐mTOR pathway protein levels, including p‐Akt, p‐4E‐BP‐1, p‐S6K and p‐S6. Altogether, these findings indicate that the 50/10 OIR protocol leads to an overall induction of autophagy in the retina that reaches its maximum at P14, to then decline until P18 when room air oxygen tension is stabilized. However, since the AMPKα/Ulk1 pathway is not the sole activator of the autophagic process, we cannot exclude that additional molecular pathways may participate in autophagy induction in the OIR rat retina.

As reported, p62 protein is degraded upon induction of the autophagic flux when the autophagosomes fuse with lysosomes.^32^ Here, a significant increase of LC3‐II was not accompanied by a decrease of p62 levels in the OIR retina. In this respect, an upregulation of LC3‐II can indicate either an activation of autophagy or an accumulation of autophagosomes. On the contrary, p62 is considered an autophagic substrate whose variation is related to changes in late autophagy stages. However, p62 does not only play a role as cargo receptor in autophagy, but is also involved in multiple other molecular pathways, including those involved in protein quality control and ubiquitination, and in inflammation.^32^, ^50^ Modulation of p62 levels can then be independent from an alteration of autophagy. Exploratory results obtained with CQ, an inhibitor of lysosomes that raises lysosomal pH and prevents the activity of lysosomal acid‐proteases, thus causing autophagosomes accumulation, indicate that neither LC3‐II nor p62 levels changed by lysosome inhibition. These data support the hypothesis of a blockade in the autophagic flux during late stages of autophagy in the OIR retina that prevents p62 degradation. Therefore, in this model the autophagolysosome formation would be limited by the availability of the terminal lysosomal system, ultimately resulting in an autophagic flux blockade. This hypothesis is supported by recent findings indicating that prolonged stresses induce an increase in autophagosome synthesis and a compromised autolysosomal activity, thus limiting the fusion of autophagosomes with lysosomes and contributing to cell damage and autophagy‐associated cell death mechanisms.^51^ In this respect, an accumulation of autophagosomes has been related to several cell death mechanisms, including necroptotic RIPK‐mediated mechanisms, which can be induced directly by the autophagy machinery.^52^

Necroptotic mechanisms have been reported to promote neuronal cell death in neurodegeneration and eye diseases.^53^ The crosstalk between autophagy and necroptosis has been the focus of multiple studies, although the mechanism is not fully elucidated. Necroptotic mechanisms can be induced upon a blockade of the autophagy flux, through a crosstalk between p62, LC3‐II and necroptosis markers, such as RIPK‐1 and RIPK‐3.^54^, ^55^ Moreover, autophagy may occur in association with necroptosis when triggered by apoptotic caspase‐mediated inhibition. At a molecular level, upon the activation of apoptosis, the RIPK activity is subject to a tight repression, through active caspase 8‐mediated cleavage.^56^ Interestingly, our results demonstrate an increased expression of LC3, RIPK‐1 and RIPK‐3 in GCL, IPL, OPL, ONL and OS layers of the OIR retina, in agreement with previous studies in rodent retinas.^26^ These findings are accompanied by a predominant expression of active caspases in retinal endothelial cells. Unlike RIPK‐1 and RIPK‐3, which were only detected in OIR retinas, the expression of active caspases was detected also in retinal endothelial cells of RA rats. It is important to highlight that at these time points the retinal vasculature in rat pups is still developing.^57^ For instance, the expression of Casp‐8 has been reported as essential for the proper postnatal retinal vascularization, contributing to the physiological vascular remodelling process, as demonstrated in developing rodent retinas.^58^ Our data indicate an activation of apoptotic processes as a consequence of the OIR protocol, since active caspase 3 expression increases in the OIR retina, concomitantly to the increase in active caspase 8 levels. In addition, we did not detect apoptotic nuclei in the studied retinal sections by TUNEL assay. The absence of nuclear DNA damage indicates that the cell death mechanisms of necrosis and apoptosis characterized in our study may be predominantly mediated by the dysfunctional autophagy, putatively associated with damaged cytoplasmic components and organelles.

In a rat model of retinal ischaemia, an inhibition of autophagy associated with retinal cell death has been shown to prevent neuronal death following injury.^37^ Following the observation that LC3 and RIPKs are co‐expressed in the same retinal layers, we evaluated if inhibition of autophagosomes nucleation with 3‐MA^59^ could decrease necroptosis in OIR retinas. 3‐MA is a non‐specific autophagic inhibitor, as this drug blocks both isoform I and isoform III of the phosphatidylinositol 3 kinase (PI3‐K). However, this issue is common among the most widely used autophagic inhibitors, which are pan‐PI3‐K inhibitors with possible off‐target effects, which could affect non‐autophagic pathways.^60^ Given that our Western blot analysis has evidenced an inhibition of LC3 lipidation mediated by 3‐MA, it is likely that the overall effect of 3‐MA in the present model targets primarily PI3‐KIII, which acts downstream of PI‐3KI. In this respect, it has been demonstrated that prolonged incubation with 3‐MA may activate autophagy in nutrient‐rich conditions, whereas during starvation (a condition reproduced in the OIR model) 3‐MA treatment leads to an autophagic blockade.^61^ Moreover, a strong Akt deactivation due to PI3‐KI blockade would lead to an enhancement in apoptosis, as reported in models of retinal diseases.^62^, ^63^ However, our Western blot data show a 3‐MA‐dependent inhibition of autophagy, whereas no effects have been observed on active caspase 3 levels. Therefore, considering the complex relationship between autophagy and apoptosis, it is likely that, in our model, the modulation of one mechanism without having effects on the other is possible, thus suggesting that 3‐MA treatment affects the activity of PI3‐KIII over that of PI3‐KI. Interestingly, our results show a notable decrease of both autophagic and necroptotic markers in several layers of OIR retinas treated with 3‐MA, yet no reduction of apoptotic markers was observed in retinal endothelial cells. Taken together, these results suggest that the OIR protocol in rats leads to an induction of autophagy‐dependent necroptotic mechanisms in retinal neurons, concomitantly with an increase of autophagy‐independent apoptotic mechanisms in retinal endothelial cells, as previously suggested.^58^

In retinal diseases, mechanisms of retinal cell death have been correlated with retinal dysfunction and visual loss.^64^ Concomitantly with reducing retinal cell death, the inhibition of autophagy has been shown to have a neuroprotective effect and to result in a partial recovery of visual impairment.^65^ By recording ERG responses to full‐field light flashed retinas, our data show that, although the treatment with 3‐MA reduces necroptosis in retinal cells, visual function is not ameliorated in OIR rats treated with the autophagy inhibitor. This is in agreement with previous findings in OIR mice, in which the inhibition of autophagy strongly reduces the autophagic flux in endothelial cells, without improving retinal function, as assessed by ERG recordings.^66^ This result could be associated with the fact that, in OIR retinas, the retinal endothelial cells are still branded by a strong activation of apoptosis. On the contrary, alternative mechanisms of autophagy‐independent cell death might be affecting the neurons’ functions, as demonstrated in the neuroretina of diabetic rats.^67^ Therefore, despite a reduction of necroptosis in retinal cells, retinal function still remains compromised due, for instance, to a dysfunction of retinal neurons involved in the generation of ERG traces.

Collectively, the present study demonstrates an activation of the autophagic pathway correlated with necroptosis in retinal cells of OIR rat pups. In this model, the stress induced by oxygen fluctuations from birth to P14 leads to a retinal energetic starvation, which results in the induction of autophagy, through an activation of the AMPKα/Ulk1 pathway. Thus, the autophagy induction initially may act as a survival attempt to restore cellular energy in the retina, yet fails due to a putative impairment of the lysosomal system. This failure leads to an accumulation of autophagosomes, which culminate in the activation of mechanisms of cell death, such as necroptosis, that further affect the retina of OIR rats.

In conclusion, the present findings contribute to deepen the understanding of the role of autophagic processes in vasculature and the neuroretina of rats exposed to the OIR protocol. Moreover, the present study supports the possibility that negative pharmacological regulation of autophagy may contribute to reduce the mechanisms of cell damage that occur in retinal cells exposed to prolonged stress, a condition paralleled in ROP‐afflicted preterm babies.

## CONFLICT OF INTEREST

The authors declare no conflicts of interest.

## AUTHOR CONTRIBUTIONS

**Noemi Anna Pesce:** Data curation (equal); Formal analysis (equal); Investigation (equal); Visualization (equal); Writing‐original draft (equal). **Alessio Canovai:** Formal analysis (equal); Investigation (equal); Visualization (equal); Writing‐original draft (equal). **Flavia Plastino:** Formal analysis (supporting); Investigation (supporting); Visualization (supporting); Writing‐original draft (supporting). **Emma Lardner:** Investigation (supporting). **Anders Kvanta:** Supervision (supporting). **Maurizio Cammalleri:** Formal analysis (supporting); Investigation (supporting); Visualization (supporting). **Helder André:** Conceptualization (equal); Funding acquisition (supporting); Project administration (equal); Supervision (equal); Writing‐review & editing (equal). **MASSIMO DAL MONTE:** Conceptualization (equal); Funding acquisition (lead); Project administration (equal); Supervision (equal); Writing‐review & editing (equal).

## Supporting information

Supplementary Material

## Data Availability

The data that support the findings of this study are available from the corresponding author upon reasonable request.
