# Supplementary material for: An imbalance in autophagy contributes to retinal damage in a rat model of oxygen‐induced retinopathy
Source: J Cell Mol Med. 2021 Oct 8;25(22):10480–93. doi: 10.1111/jcmm.16977 (PMC8581343; doi:10.1111/jcmm.16977)
Supplement: Supplementary file 1 — Supplementary Material [file JCMM-25-10480-s001.pdf]

An imbalance in autophagy contributes to retinal damage in a rat model of oxygen-induced retinopathy

Noemi Anna Pesce[1,2,§], Alessio Canovai[1,§], Flavia Plastino[2], Emma Lardner[2], Anders Kvanta[2], Maurizio Cammalleri[1], Helder André[2,*], Massimo Dal Monte[1,*]

[1]Department of Biology, University of Pisa, Pisa, Italy.

[2]Department of Clinical Neuroscience, Division of Eye and Vision, St Erik Eye Hospital, Karolinska Institutet, Solna, Sweden.

[§]Equal contributing authors

*Equal contributing senior authors

SUPPLEMENTARY MATERIALS AND METHODS

Chloroquine and 3-MA pharmacological treatments

To determine the autophagic flux, 12 OIR rat pups were treated with an intraperitoneal injection of 60 mg/kg of chloroquine diphosphate (CQ; ab1421116; Abcam, Cambridge, UK) diluted in normal saline solution. The injection was performed either at P14 (6 rats) or P18 (6 rats), 6 h before sacrifice. An equal number of OIR rats were treated with a daily intraperitoneal injection of 3 mg/kg of 3-MA (cat. no. sc-205596; Santa Cruz Biotechnology; Dallas, TX, USA) in normal saline solution, from birth to P14 (6 rats) or P18 (6 rats). As controls, 12 OIR rat pups were injected with an equal volume of vehicle until P14 (6 rats) or P18 (6 rats).

TUNEL assay

Terminal deoxynucleotidyl transferase-mediated dUTP nick-end labeling (TUNEL) assay was performed on retina sections from RA, OIR and OIR + 3-MA rats at both P14 and P18. Click-iT™ Plus TUNEL Assay For In Situ Apoptosis Detection, Alexa Fluor™ 594 dye kit (Thermo Fischer Scientific, USA) was used according to the manufacturer ’s protocol. A positive control was ran on RA P14 retina section, using DNase I (Cat. No. 18047-019; Thermo Fisher Scientific) diluted in DNase I Reaction Buffer (20 mM Tris-HCl, pH 8.4; Bio-rad laboratories), 2 mM MgCl2 (CAS 7791-18-6, Merck Group, Darmstadt, Germany), 50 mM KCl (CAS 7447-40-7, Merck Group) to induce

DNA fragmentation. Images were acquired with an Axioskop 2 plus fluorescence microscope with the AxioVision software (Zeiss, Gottingen, Germany).

Supplementary Figure S1

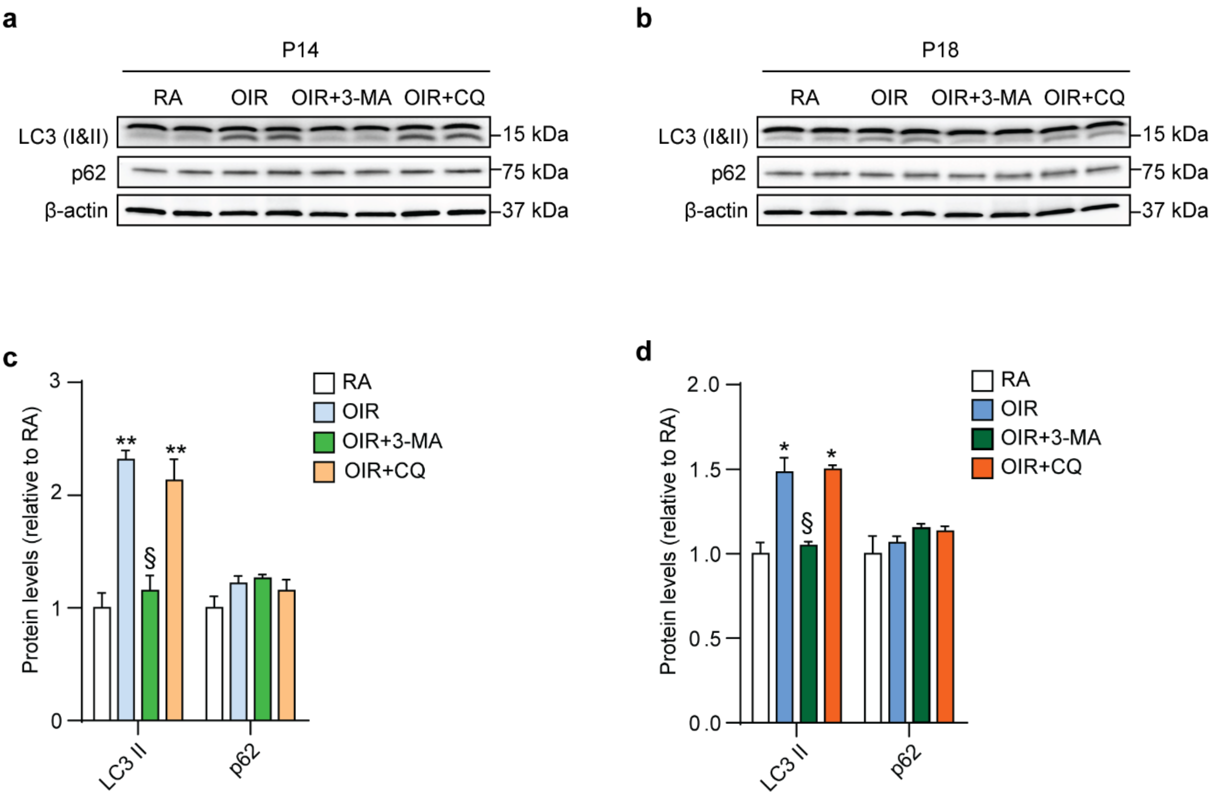

Supplementary Figure S1. a, b Representative immunoblots of LC3-I, LC3-II, p62 and β-actin (loading control) in RA, OIR, OIR + 3-MA and OIR + CQ rats at P14 (**a**) and P18 (**b**). **c, d** Densitometric analysis of LC3-II and p62 levels in RA, OIR, OIR + 3-MA, OIR + CQ rats at P14 (**c**) and P18 (**d**). Data are plotted as mean ± SEM. Differences between groups were tested for statistical significance using one-way ANOVA followed by Bonferroni's multiple comparisons post-test (n=6 animals per group). *$p < 0.05$, **$p < 0.01$ vs respective P14 and P18 RA; $^{\S}p < 0.05$ vs respective P14 and P18 OIR.

Supplementary Figure S2

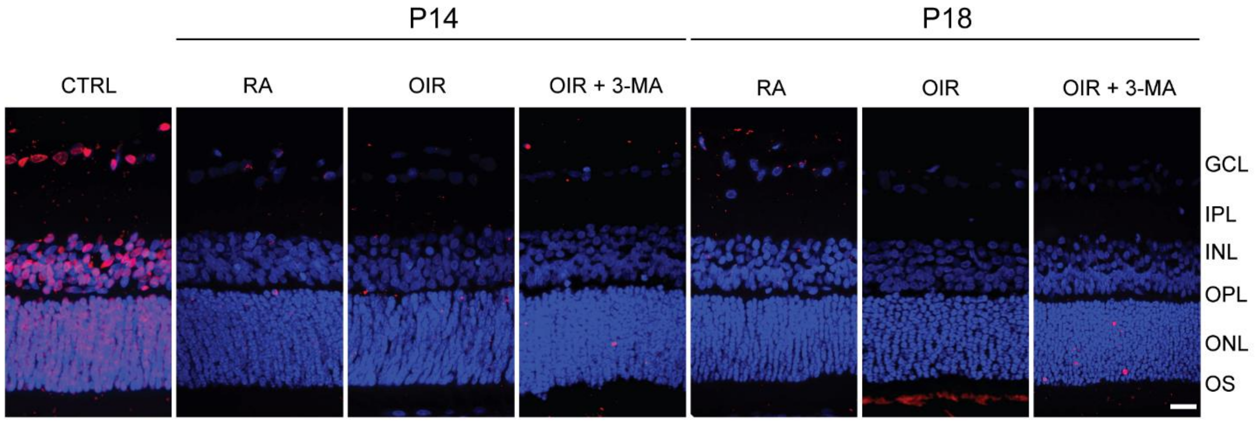

Supplementary Figure S2. Detection of apoptotic cells by TUNEL assay in RA, OIR and OIR+3-MA rat retinas at P14 and P18. TUNEL-positive cells were identified with red fluorescence, while the nuclei were counterstained with Hoechst (blue). No TUNEL-positive cells were found in RA, OIR and OIR+3-MA rat retinas at P14 and P18, compared to the positive control (CTRL). GCL, ganglion cell layer; IPL, inner plexiform layer; INL, inner nuclear layer; OPL, outer plexiform layer; ONL, outer nuclear layer; OS, outer segments of photoreceptors. n=6 sections per group. Scale bar = 50 µm.
